# High-resolution mass spectrometry for extended PFAS surveillance in food: combining suspect and non-targeted approaches

**DOI:** 10.1016/j.fochx.2025.102843

**Published:** 2025-07-29

**Authors:** Cassandre Jeannot, Nicolas Macorps, Ahmed Amziane, Bruno Le Bizec, Julien Parinet, Gaud Dervilly

**Affiliations:** a Oniris, INRAE, LABERCA, 44300 Nantes, France; b ANSES, Laboratory for Food Safety, Pesticides and Marine Biotoxins Unit, 14 rue Pierre et Marie Curie, F-94701 Maisons-Alfort, France

**Keywords:** Fluorinated contaminants, Environmental hotspots, Chemical surveillance, Food safety monitoring, Emerging pollutants, Exposure variability

## Abstract

*Per*- and polyfluoroalkyl substances (PFAS) are persistent, potentially harmful synthetic chemicals. While they can accumulate in foodstuffs, current monitoring often targets only a few compounds, likely underestimating dietary exposure. In this study, 58 food samples from Europe and North Africa—including commercial products and items from known European contamination hotspots—were analyzed using a validated high-resolution mass spectrometry workflow combining suspect screening (SS) and non-targeted screening (NTS). Seventeen PFAS were confirmed through SS, with up to 15 different PFAS in fish samples from hotspots. While NTS revealed four additional fluorinated substances: Perfluoropropanoic acid (PFPrA) detected in 48 % of samples, 6:2 Fluorotelomer sulfonic acid (6:2 FTS), Fipronil, and Fipronil sulfone. These results highlight the geographical variability of PFAS contamination in food and demonstrate the value of combined SS/NTS approaches in identifying both known and emerging PFAS, supporting more comprehensive, regulation-aligned risk assessments.

## Highlights

• SS/NTS approaches reveals 21 different fluorinated compounds in food samples.
• PFPrA identified for the first time in commercial foods.
• 6:2 FTS, Fipronil and Fipronil sulfone are observed in eggs from hotspot areas.
• Hotspot samples support prioritization of emerging PFAS signals.
• HRMS-based strategy broadens PFAS detection for more accurate risk assessment.

## Graphical abstract

**Figure:**
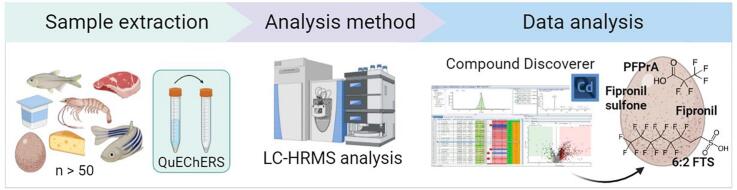

## 1 Introduction

*Per*- and polyfluoroalkyl substances (PFAS) have been used since the 50s and are widely applied in various industrial and commercial products owing to their unique properties (e.g. excellent thermal and chemical stability) (Glüge et al., 2020). According to the Organization for Economic Co-operation and Development (OECD) definition, PFAS are a large group of synthetic organic chemicals containing at least one fully fluorinated methyl or methylene carbon atom (Wang et al., 2021). The OECD's broadening of the definition of PFAS has made it possible to include a greater number of substances, including new-generation alternatives, but has also led to the inclusion of substances with different uses, such as fluorinated pesticides (Wang et al., 2021). More recently, in order to simplify and include even more compounds in this group, the Environmental Protection Agency (EPA) has introduced a new definition of PFAS that “*removes the requirement that the structure is entirely aliphatic, and only requires that the minimal fully fluorinated methyl or methylene group is saturated and aliphatic*” (Gaines et al., 2023). Certain compounds within this broad class of chemicals are considered persistent organic pollutants (POPs), due to their persistence, bioaccumulation, and toxic properties leading to the inclusion of some of them (such as perfluorooctane carboxylate (PFOA), perfluorooctane sulfonate (PFOS) and perfluorohexane sulfonate (PFHxS)) in the annexes of the Stockholm Convention. They are further associated with numerous health risks, and recently, the International Agency for Research on Cancer (IARC) and the Cancer Agency of the World Health Organization (WHO) have highlighted the carcinogenicity of PFOA to humans, and the possible carcinogenicity of PFOS to humans (Zahm et al., 2024). The growing concern over PFAS contamination in food stems from their persistence and potential to bioaccumulate, leading to serious health risks such as immunosuppression, reduced vaccine efficacy. These compounds often escape detection due to limitations in current regulatory frameworks and food safety monitoring programs, which typically target only a narrow subset of PFAS. This gap underscores the urgent need for more comprehensive surveillance and risk-assessment strategies to ensure effective public health protection (Government of France, 2024).

Commonly referred to as legacy PFAS, PFOA and PFOS are long-chain compounds containing eight carbon atoms (C8). Following restrictions on their use, shorter-chain PFAS (C < 8), also known as short-chain PFAS (scPFAS) have been increasingly used as alternative to long-chain PFAS. However, these new PFAS are no less toxic (Zheng et al., 2023), and PFHxS was included in the Stockholm Convention 13 years after the first two. Despite assumptions related to their smaller molecular size, recent studies have demonstrated that scPFAS are equally persistent and bioaccumulative. Moreover, they exhibit long-range environmental transport potential and have already been detected across various environmental compartments (e.g. indoor dust, air, drinking water, soil, sediment, and aquatic systems) (Zheng et al., 2023). As a result, these substances are ubiquitous environmental contaminants, commonly detected in humans and food (Ng et al., 2021) one of the primary pathways of exposure to PFAS (EFSA, 2020). Among food categories, fish, seafood and eggs are regarded as the primary contributors to PFAS exposure through the human diet (Chiumiento et al., 2023; EFSA, 2020).

In 2020, the European Food Safety Authority (EFSA) revised the Toxicological Reference Values for PFAS significantly downwards, and set a tolerable weekly intake (TWI) for the sum of the four (PFOA, PFOS, perfluorononanoic acid (PFNA) and PFHxS) at 4.4 ng/kg bw/week. As a result, maximum levels in certain foodstuffs have been defined for these 4 compounds (European Commission, 2022b). This shift in PFAS risk assessment and risk management in food has been accompanied by efforts to develop more sensitive and specific analytical methods to improve associated monitoring (EFSA, 2020; European Commission, 2022a).

However, and although a few other PFAS are also listed as of interest to be monitored in foods (around twenty), it has to be said that this effort only concerns a minor fraction of the thousands of compounds meeting the PFAS definition. This number is rising steadily, from over 4700 in 2018 (Wang et al., 2021) to over 7 million PFAS reported in PubChem, 5 years later (Schymanski et al., 2023).

To address the challenge of expanding PFAS surveillance, a shift towards broader analytical coverage was necessary. Recent non-targeted profiling technologies facilitate the global evaluation of the chemical exposome, enabling the detection of a wider range of chemicals. These strategies rely on three main approaches to broaden the PFAS chemical space: non-specific sample preparation methods, such as QuEChERS (Quick, Easy, Cheap, Effective, Rugged, Safe) due to its simplicity and efficiency, high-resolution mass spectrometry (HRMS) data acquisition, and data processing techniques that prioritize fluorinated signals.

HRMS has been increasingly employed to identify novel PFAS (Feng et al., 2024; Ren et al., 2022; Wang et al., 2022). There are two primary strategies for PFAS identification: suspect screening (SS) and non-target screening (NTS). SS is the most straightforward approach, especially when reference MS/MS (tandem Mass Spectrometry (MS^2^)) spectra are available (Diallo et al., 2023; Makni et al., 2024). In contrast, NTS is essential for identifying novel PFAS when no suspect list exists, and it relies on fragment-based and/or homologue-based screening. HRMS plays a crucial role by providing accurate mass data vital for identifying unknown contaminants. Since PFAS typically occur in homologous series, HRMS data, combined with homologue patterns or mass defects, can help identify PFAS homologues, distinguished by their accurate mass, elution order, and characteristic patterns (e.g., CF_2_, C_2_F_4_, CF_2_O). Visualization through a CF_2_-based Kendrick mass defect (KMD) plot facilitates identification, as compounds from the same class align horizontally (Liu et al., 2015; Ren et al., 2022). For HRMS data processing, software tools such as Compound Discoverer (CD) and FluoroMatch are commonly used, offering high-performance solutions for signal prioritization and identification of perfluorinated compounds (Ogunbiyi et al., 2023; Stroski et al., 2024).

This broader analytical approach supports improved risk assessment and breaks with conventional targeted approaches. Our previous work (Jeannot et al., 2025) demonstrated as a proof of concept the effectiveness of a QuEChERS / LC-HRMS (Liquid Chromatography coupled to HRMS) non-targeted workflow in identifying a previously unlisted PFAS in an egg sample, proposing a high-performance strategy to expand PFAS analyzed in foodstuffs. The objective of this study is to apply a combined SS/NTS strategy to a broad range of food samples representative of the European diet, while also extending the investigation to North Africa. PFAS contamination in foodstuffs is well-documented across Europe, particularly in fish and seafood. In this context, it is relevant to explore whether similar contamination patterns exist in other regions. Preliminary data from Algeria (Amziane et al., 2022) also indicate elevated PFAS levels in fish, suggesting potential parallels in environmental and dietary exposure. To enrich the continental-scale analysis, we strategically included a targeted set of food samples collected from private individuals living in well-documented contamination hotspots across Europe. These high-exposure samples were selected not only to evaluate the applicability and robustness of the SS/NTS approach under more extreme conditions, but also to inform signal prioritization strategies. This cross-continental approach with real-life scenarios aims to better characterize regional PFAS contamination patterns, ultimately supporting more comprehensive risk assessment efforts.

## 2 Materials and methods

### 2.1 Chemicals and reagents

Sodium Chloride (NaCl) was obtained from Merck (Darmstadt, Germany), HF Bond Elut – C_18_ was obtained from Agilent Technologies (Santa Clara, CA, USA) and Magnesium Sulfate (MgSO_4_) from ThermoScientific. Acetonitrile (ACN) was from Honeywell, and formic acid (FA) and supelclean ENVI-Carb SPE (solid-phase extraction) tube from Supelco (Sigma-Aldrich, Saint-Quentin Fallavier, France). Ultrapure water (>14 MΩ.cm) and water for LC-HRMS from VWR (Radnor, PA, USA) were used.

For SS analysis, 28 PFAS compounds along with 15 labelled reference compounds were used as standards and internal standards (Jeannot et al., 2025). At a later stage, to improve and expand the non-targeted method, additional PFAS standards (17 non-labelled PFAS and 5 isotopically labelled PFAS) were acquired, thereby broadening the analytical scope. All the PFAS standards were sourced from Wellington Laboratories and Cambridge Isotope Laboratories, as detailed in Table S1. A ‘PFAS mix’ solution containing the 45 analytical standards, was thus prepared in methanol at a concentration of 0.01 ng/μL for each compound. A separate solution containing the 20 labelled PFAS, including 5 additional compounds purchased, used as internal standards was also prepared in methanol at 0.01 ng/μL for each compound. Additionally, a solution of 13C8 PFOS was prepared in methanol (0.01 ng/μL) as an external standard.

### 2.2 Investigated samples

**Fig. 1:**
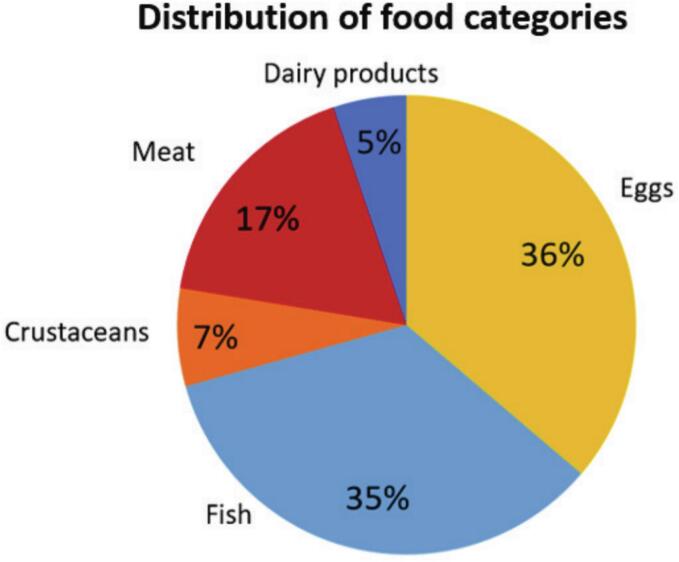
Distribution of the 58 food samples analyzed, grouped by food category (*n* = 5), regardless of their origin (France, Algeria, or European hotspots).

This study focused on foods known to contribute to consumer exposure to PFAS. **France.** A set of twenty samples whose origin was described in (Jeannot et al., 2025) was further characterized: 3 eggs, 4 crustaceans, 5 fish, 5 meat products, and 3 dairy products. **Algeria.** A sub set of 20 samples previously characterized using a targeted PFAS approach has been selected as follows: 5 eggs, 10 fish, and 5 meat samples (Amziane et al., 2022). **European contamination hotspots.** Eighteen samples were collected from private individuals living in known PFAS contamination hotspots in Europe: 13 eggs and 5 fish. A total of 58 samples, collected in Europe and North Africa between 2019 and 2021, were thus investigated, and were stored at −20 °C until analysis. Fig. 1 shows the distribution of the 58 food samples by food category, highlighting an apparent over-representation of eggs and fish due to the inclusion of additional samples from individuals living in PFAS-contaminated European hotspots. Detailed information on the samples analyzed, including their origin, is provided in Table S2.

### 2.3 Sample preparation

All samples were extracted using the QuEChERS protocol described previously (Jeannot et al., 2025). Briefly, samples (150 mg) were spiked with internal PFAS standards solution (1 ng.g^−1^ wet weight (w.w)), and extracted with H_2_O, and ACN containing 1 % FA. Then, 600 mg NaCl were added and the sample were stored overnight at −20 °C. Supernatants were evaporated under nitrogen to 1 mL, further cleaned-up by dispersive-SPE (d-SPE) with anh. MgSO_4_, C_18_ and GCB, and filtered through a 0.22 μm nylon syringe filters. Extracts were dried using nitrogen, reconstituted in MeOH / H_2_O, and passed through an Omega 10 kDa membrane into a vial for LC-HRMS analysis.

To assess background PFAS contamination during extraction and analysis processes, an extraction blank sample, without internal standards, was prepared and analyzed identically to the samples.

### 2.4 Data acquisition and processing

The extracts were analyzed by LC-HRMS for suspect screening (SS) of PFAS for which standards are available (*n* = 28), and for non-targeted screening (NTS) to identify other potentially present fluorinated substances in the sample.

An Ultimate 3000 UHPLC pumping system coupled to an Orbitrap Q-Exactive mass spectrometer (Thermo Scientific®, Bremen, Germany) operated in negative electrospray ionization (ESI) mode was used in both full scan and DIA modes. The LC-ESI(−)-HRMS conditions, including the chromatographic column, the phase mobile composition, gradient and some other mass parameters, were previously described (Jeannot et al., 2025). Briefly, chromatographic separation was performed on a reverse phase C18-column (Hypersil Gold, 100 mm × 2.1 mm i. d., 1.9 μm particle size) equipped with a pre-filter. The total cycle time was 20 min with water 5 mmol.L^−1^ ammonium formate (A) and ACN (B). The injection volume was set to 5 μL, and the elution gradient was as follows: 0–0.5 min, 5 % B; 0.5–8 min, 5–100 % B; 8–14 min, 100 % B; 14–18 min, 100–5 % B; 18–20 min, 5 % B. The temperatures of the column oven and the sample trays were set at 40 °C and 4 °C, respectively.

#### 2.4.1 Suspect screening

In order to identify PFAS included in the database, mass spectra were acquired in full scan mode with a scan range from 65 to 975 *m*/*z* at a resolution of 70,000. Some other parameters are detailed in Section 2.4.2. Data acquisition was performed by Xcalibur software. SS data analysis was performed using Skyline, a user-friendly open-source software package (Adams et al., 2020; MacLean et al., 2010), for processing raw files acquired in full scan mode and for peak integration, as mentioned in our previous work (Jeannot et al., 2025).

#### 2.4.2 Non-targeted screening

• Initial Data Processing and Feature Extraction

The full scan raw data were processed using Compound Discoverer 3.3 software to extract candidate compounds, providing information on retention time (RT) and m/z. These candidates were classified by food type and compiled into inclusion lists.

• Data-Independent Acquisition (DIA) for Fragmentation

A second injection was performed to acquire MS/MS spectra in DIA mode using inclusion lists. Acquisition parameters were optimized for efficient fragmentation of up to 30 candidates, with specified source conditions and collision energies as follows: spray voltage 3 kV; s-lens radio frequency 40 AU; sheath gas flow 55 arbitrary units (AU); auxiliary gas flow rate 10 AU; capillary temperature 350 °C; auxiliary gas temperature 300 °C. A stepped normalized collision energy of 10, 30, and 90 electronvolts (eV) was employed. For the first injection, the automatic gain control (AGC) target and the maximum injection time (IT) were set at 1.10^6^ and 100 ms, respectively. For the second injection, the AGC target and the maximum IT were set at 2.10^5^ and set to auto, respectively. Data acquisition was performed by Xcalibur software.

• Compound Discoverer Workflow

The DIA data were processed using a modified Compound Discoverer workflow adapted from (Kaufmann et al., 2022; Sanchez & Tautenhahn, 2023) (Fig. S1). Key nodes included: Input Files → Select Spectra → Align Retention Times → Detect Compounds → Group Compounds. The default settings were used for the Select Spectra and Align Retention Times nodules. The following parameters were used in the Detect Compounds nodes:

(1) General settings.—Mass Tolerance = 5 ppm, Min. Peak Intensity = 1000, Min. # Scans per Peak = 5, Use Most Intense Isotope Only = True
(2) Trace detection.—Max. Number of Gaps to Correct = 2, Min.Number of Adjacent Non-Zeros = 2
(3) Peak detection.—Chromatographic S/N Threshold = 3, Remove Baseline = False, Gap Ratio Threshold = 0.35, Max.Peak Width [min] = 1, Min. Relative Valley Depth = 0.1
(4) Compound detection.—Ions = [M-H]-1; [M + H] + 1; [M + Na] + 1; [M + NH_4_] + 1, Remove Singlets = True

• Annotation and Library Matching

CD was used to conduct NTS for novel PFAS compounds through the ‘Search ChemSpider’ and ‘Search mzCloud’ functions, which allow access to online spectral libraries. In addition, CD facilitated the identification of potential PFAS compounds using the ‘Search Mass Lists’ and ‘Search mzVault’ functions, which rely on in-house spectral libraries. These libraries incorporated multiple databases for feature matching, including the US EPA Comptox (PFASTRUCT) database with 10,737 chemicals and the NIST PFAS Suspect List with 4951 chemicals. In addition to the PFAS already included in our database (*n* = 28) and used for SS, 17 additional non-labelled PFAS and 5 isotopically labelled PFAS compounds were acquired to cover a broader range of physicochemical properties and to further optimize the non-targeted workflow. An in-house mass list database was also established, comprising 45 PFAS and 20 labelled ones.

• Batch Processing and Blank Subtraction

To control for contamination and improve reliability, blank subtraction was manually performed. Extracts from different food categories were processed within the same batch.

Prioritization strategy for identifying novel PFAS compounds

• Feature Prioritization and Filtering

A custom post-processing script was implemented to calculate key descriptors (e.g., mass normalized to the number of carbon atoms (m/C), mass defect (md) normalized to the number of carbon atoms (md/C), estimated F) for prioritizing fluorinated features, following (Kaufmann et al., 2022; Sanchez & Tautenhahn, 2023; Stroski et al., 2024). To reduce the number of features, the m/C versus md/C space and the KMD plot were represented using the functions included in CD software. The m/C was determined by dividing the measured mass of the monoisotopic peak by the estimated number of carbon atoms (C).

• Confidence Level Assignment

Then, an additional data processing filter, based on the parameters described by (Sanchez & Tautenhahn, 2023), was optimized to retain both PFAS with available standards in our laboratory and other relevant candidates. Only features with the following criteria are investigated: −0.10 ≤ md/C ≤ 0.15; m/C > 35; or maxF(CS) ≥ 3; maxF(ML) ≥ 3; F ≥ 3; Checked: true.Further, additional rules based on experience with PFAS and NTS analysis, such as the inconsistency for a suspected long-chain PFAS compound to exhibit a short retention time, were applied to reduce the number of features by selecting only the most probable compounds (Chu & Letcher, 2024). These different feature prioritization steps allowed the creation of a list of suspect PFAS. Confidence levels for compounds identification were assigned according to the levels of PFAS identification proposed by (Charbonnet et al., 2022), which is based on Schymanski's classification (Schymanski et al., 2014).

## 3 Results and discussion

The analytical strategy was applied to a set of food samples (*n* = 58), selected for their known contribution to dietary exposure to PFAS. The objective was twofold: first, to characterize the occurrence of PFAS included in the suspect list based on available reference standards (SS) and second, to apply a NTS workflow to detect additional fluorinated features potentially present in the samples. The sampling strategy was designed to cover two distinct geographical regions—France and Algeria—in order to provide comparative insights into their respective contamination profiles, and to include additional samples from European hotspots known to be highly contaminated with PFAS. After extraction by the QuEChERS method described in Section 2.3., the extracts were analyzed by the LC-ESI(−)-HRMS method described in Section 2.4. using full scan and DIA acquisition modes.

### 3.1 Suspect screening analysis

The availability of 28 PFAS reference standards initially included in our database made it possible to investigate their occurrence in the foods studied using suspected approach. The data acquired from these samples were processed by Skyline software, as described in Section 2.4.1. Originally developed for targeted proteomics, Skyline has proven to be a valuable tool in small molecule analysis workflows, particularly for monitoring signal intensities, assessing chromatographic peak quality, and ensuring the reliability of extracted ion chromatograms (XICs). This allowed for a more accurate inspection of the data, especially for known PFAS, and provided an additional level of confidence in the overall feature extraction and identification strategy.

**Fig. 2:**
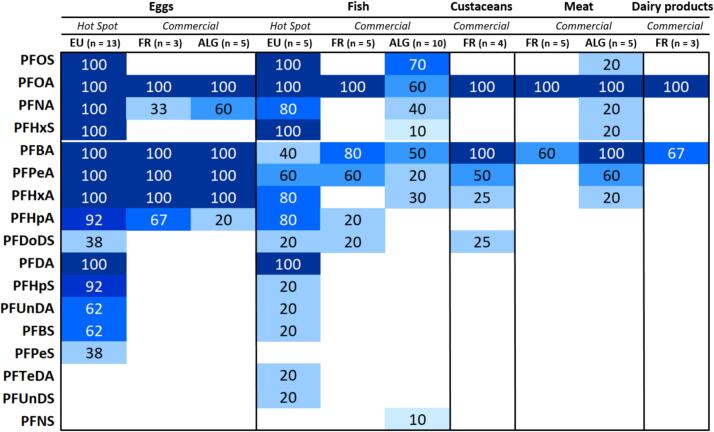
Detection frequencies (%) of 17 PFAS identified from an initial suspect screening database of 28 compounds, across five food categories (*n* = 58 samples), by geographical origin: France (FR), Algeria (ALG), and European Union (EU) hotspot samples.

As a first overview, 17 out of the 28 PFAS included in the suspect database could be unambiguously identified across the full set of 58 food samples. All identified compounds were indeed assigned the highest level of confidence (Level 1), based on the PFAS identification confidence scale proposed by (Charbonnet et al., 2022). Fig. 2 presents the detection frequency (in %) of the 17 identified PFAS across various food categories (eggs, fish, shellfish, meat, dairy products), with comparisons between commercial samples from Europe and North Africa—specifically France and Algeria. The dataset also includes a subset of European samples collected from individuals residing in known contamination hotspots, allowing for a more detailed assessment of PFAS occurrence in high-exposure contexts.

In commercial eggs, samples from both North Africa (Algeria) and Europe (France) exhibited comparable PFAS occurrence profiles, with compounds such as PFOA, perfluorobutanoic acid (PFBA), perfluoropentanoic acid (PFPeA), and perfluorohexanoic acid (PFHxA) consistently detected in a large proportion of samples from both regions. However, the subset of European Union (EU) samples collected from individuals living in identified contamination hotspots showed significantly higher detection frequencies across a broader spectrum of PFAS—including PFOS, PFNA, PFHxS, perfluoroheptanoic acid (PFHpA), perfluorodecanoic acid (PFDA), and perfluoroheptane sulfonic acid (PFHpS)—indicating the influence of localized contamination sources on the PFAS profile in this food matrix.

In commercial fish, a rather similar PFAS profile was observed in both French and Algerian samples, with PFOA being the most frequently detected compounds. Again, fish samples collected from EU hot spots exhibited elevated detection frequencies across several PFAS, including PFOA (100 %), PFBA (100 %), PFHxA (80 %), and PFHpA (80 %), pointing to intensified contamination levels in specific areas.

In crustaceans, PFOA and PFBA appeared as the most prevalent compounds in French samples (100 %), followed by PFPeA, which was also detected, albeit at lower frequencies. These results suggest a more limited PFAS profile in this matrix, but nonetheless indicate that certain legacy PFAS, such as PFOA, remain detectable in seafood products.

In meat, high detection frequencies were observed for PFOA in both French and Algerian samples, with PFBA also commonly detected, indicating that this matrix represents a notable route of PFAS exposure regardless of origin.

In dairy products, PFBA and PFOA were the only compounds consistently detected, reflecting a narrower contamination profile for this food category.

Overall, the heatmap reveals a recurring presence of both short-chain PFAS (e.g., PFBA, PFPeA) and legacy compounds (e.g., PFOA, PFOS) across all food categories. Notably, the European hotspot samples exhibited a broader and more intense PFAS contamination profile, emphasizing the role of geographical variability and the influence of localized contamination sources in shaping dietary exposure to PFAS.

Regardless of food category or geographic origin—whether from Europe or North Africa—PFOA, PFBA, PFPeA, and PFHxA emerged as the most frequently detected compounds. These four PFAS, along with others among the 17 identified, have previously been reported in a variety of food samples in earlier studies (Chiumiento et al., 2023; Genualdi et al., 2022; Rushing et al., 2023). Among these, only PFOA is one of the four PFAS included in the tolerable weekly intake (TWI) of 4.4 ng/kg body weight established by (EFSA, 2020), and also currently subject to regulatory limits in certain food items (European Commission, 2022b). Nevertheless, the other PFAS identified in this study are among those recommended for monitoring and further investigation (European Commission, 2022a).

Suspect analysis also confirmed the hotspot origin of European samples (Fig. 2). Compared to eggs and fish from the market, hotspot samples showed a significantly higher number and frequency of PFAS identifications. These results are not surprising, as samples collected from known contamination hotspots are expected to reflect more intense and diverse PFAS contamination. For example, 14 PFAS were detected in hotspot egg samples versus 6 in market ones, and 15 in hotspot fish samples compared to only 5 from the market. Detection frequencies were also notably higher, such as PFNA being found in 100 % of hotspot egg samples versus just 33 % in those from the market. These observations confirm that hotspot sampling provides a valuable opportunity to detect a broader range of PFAS, including those not routinely monitored, making it a relevant complement to non-targeted screening strategies.

These findings demonstrate the relevance and added value of integrating SS HRMS workflows into routine food safety monitoring. The approach not only enables the detection of a wide spectrum of PFAS—including both legacy and emerging compounds—but also provides a detailed picture of contamination profiles across different food categories and geographical origins.

### 3.2 Non-targeted screening analysis

In addition to suspect screening, a NTS approach was implemented to broaden the scope of PFAS detection beyond compounds with available analytical standards. This strategy enables the exploration of unknown or emerging fluorinated substances by capturing all detectable features associated with fluorine-specific signatures. The data processing workflow was first optimized, enabling the identification of additional fluorinated compounds, and was finally applied to the characterization of commercial food samples.

#### 3.2.1 Optimizing the processing of data acquired in non-targeted mode for the detection of fluorinated signals

In non-targeted analytical workflows, data processing remains a critical and challenging step, as it is essential for effective feature selection and compound identification while limiting false positives. Data were initially processed using CD software with default parameters, as described by (Kaufmann et al., 2022) and (Sanchez & Tautenhahn, 2023). A PFAS mixture (20 isotopically labelled at 0.007 ng/μL and 45 non-labelled at 0.1 ng/μL) was analyzed to evaluate this workflow. Skyline software (Adams et al., 2020; MacLean et al., 2010) was also used to evaluate and compare the results obtained with CD.

This preliminary evaluation demonstrated that, even without specific parameter optimization, Skyline successfully detected signals corresponding to all PFAS compounds analyzed (*n* = 45 native +20 labelled). In contrast, data reprocessed using Compound Discoverer identified only 12 labelled PFAS and 39 native PFAS, thereby revealing a suboptimal parameterization of the initial CD workflow.

An important aspect of the workflow optimization involved refining the mass defect filtering parameter, which plays a key role in suspect screening. As initially configured according to (Sanchez & Tautenhahn, 2023), this filter excluded compounds based on their mass defect, retaining only PFAS with a negative mass defect (md/C < −0.0007). However, this stringent criterion was found to be overly restrictive, potentially discarding relevant PFAS candidates.

To broaden the chemical space and include PFAS with positive or neutral mass defects, the filter range was adjusted to −0.10 < md/C < 0.15, in line with data from (Wang et al., 2021), which showed that 86 % of PFAS fall within this interval. This adjustment significantly improved the inclusion of relevant features during data processing.

As a result of this optimized configuration, all 45 native PFAS were successfully detected, since six PFAS compounds previously excluded due to their positive mass defect were now included (3-Perfluoropropyl propanoic acid (FPrPA), 3-Perfluoropentyl propanoic acid (FPePA), 3-Perfluoroheptyl propanoic acid (FHpPA), 6:2 fluorotelomer sulfonamidopropyl betaine (6:2 FTAB), *N*,*N*-dimethyl-3-((perfluorohexyl)ethylsulfonyl)aminopropanamine oxide (6:2 FTAA-Ox), and 2-(N-ethylperfluorooctanesulfonamido) acetic acid (N-EtFOSAA)). Furthermore, three additional labelled PFAS (d3-N-MeFOSAA (2-(*N*-methylperfluorooctanesulfonamido) acetic acid), d5-N-EtFOSAA, and 13C4–6:2 diPAP (.

**Fig. 3:**
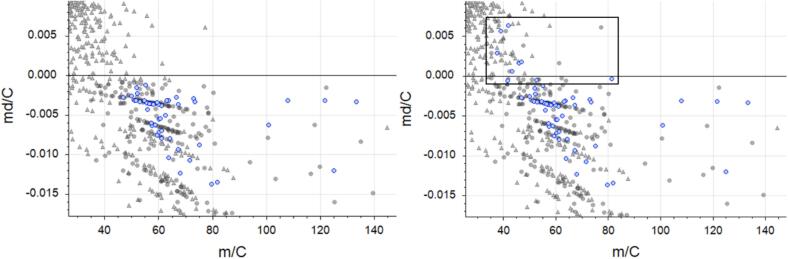
md/C vs m/C representation of the PFAS standard solution (blue circles) analyzed by LC-(ESI-)-HRMS and processed in Compound Discoverer (CD) software using the additional filter as initially defined in the literature (Kaufmann et al., 2022) (left) and after applying the optimized filter parameter developed in this study (right). (For interpretation of the references to colour in this figure legend, the reader is referred to the web version of this article.)

Bis[2-(perfluorohexyl)ethyl] Phosphate)) were also identified (Fig. 3).

Nevertheless, the identification of isotopically labelled PFAS remains challenging due to their low isotopic signal intensity, the frequent absence of a carbon count (C = 0), and their lack of representation in reference databases. For instance, fully 13C-labelled standards such as 13C4-PFBA cannot be processed via isotopic pattern recognition (Chu & Letcher, 2024).

Overall, the new mass defect filter parameters allowed the detection of 15 out of 20 labelled PFAS and all 45 native PFAS, significantly enhancing the coverage of the PFAS chemical space and improving the robustness of the suspect screening workflow (Fig. 2).

#### 3.2.2 Identification of additional fluorinated compounds in hot spot samples through non-targeted screening

**Fig. 4:**
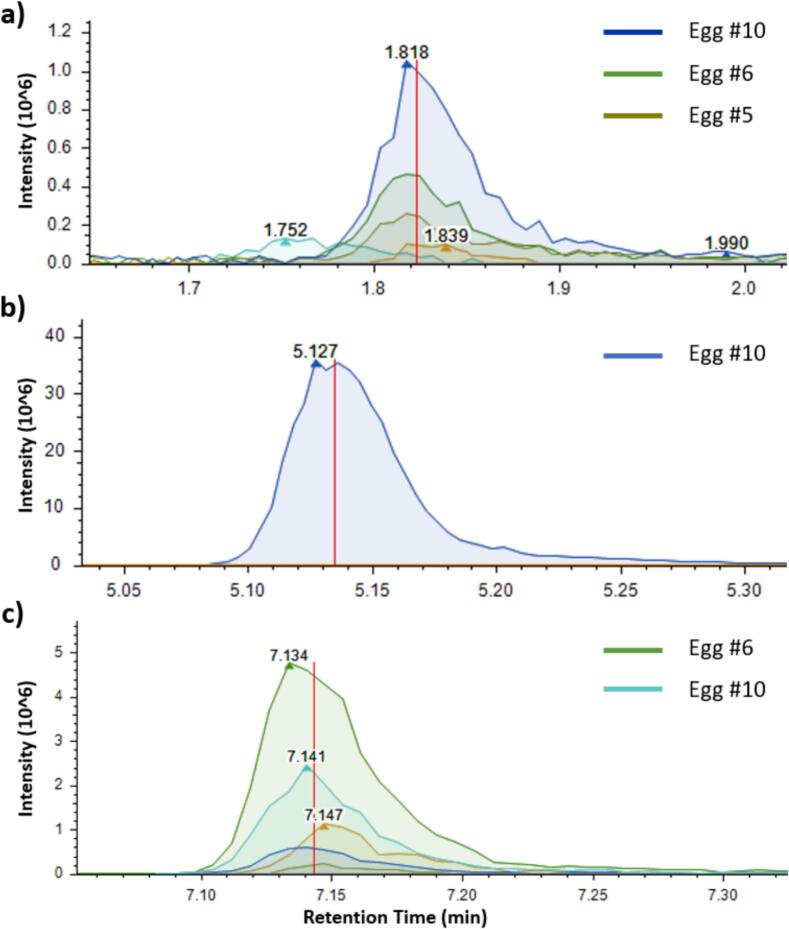
Chromatographic profile of (a) PFPrA in three egg samples, (b) 6:2 FTS in an egg sample, and (c) Fipronil sulfone in two egg samples from hotspots, analyzed by LC-HRMS using the non-targeted workflow developed in this study. Signals processed with Compound Discoverer.

Given the constraints associated with the limited availability of reference standards, non-targeted analysis offers valuable potential to uncover additional PFAS that may be present in food. The SS strategy proved effective in highlighting the most contaminated samples—designated as hotspots—which were then prioritized as key matrices for deeper non-targeted investigations. This approach focus non-targeted efforts on informative samples (Fig. 4). The acquired data were processed using the optimized CD workflow, reducing neatly 10,000 features to approximately 30 suspect fluorinated compounds for DIA confirmation. Manual refinement further filtered these based on known PFAS characteristics.

Four fluorinated compounds, which had not been previously identified through the SS approach, were unequivocally confirmed at level 1 (Schymanski et al., 2014)).

**Fig. 5:**
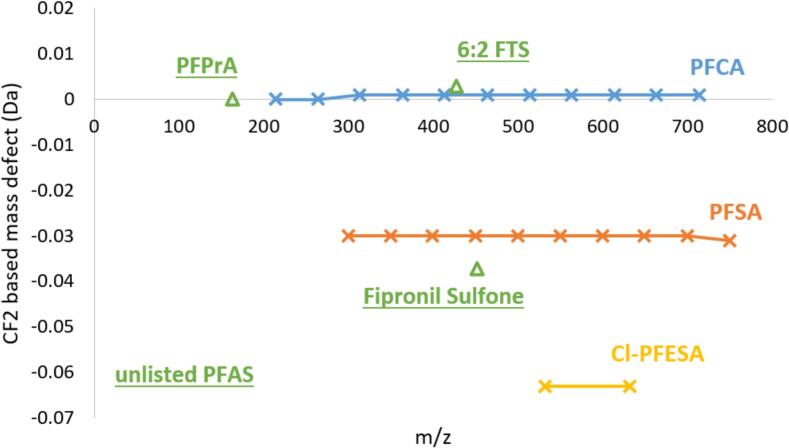
Kendrick mass defect for CF2 repeating unit vs *m*/*z* of certain PFAS standard families listed in our internal database and classified by family: PFCA (*n* = 11), PFSA (*n* = 10) and Cl-PFESA (*n* = 2). Homologous series are identified by shifts in the x-axis divisible by 49.9968 (CF2) and the same CF2 normalized mass defect. In green, three suspect PFAS signals highlighted by NTS data processing (in green). (For interpretation of the references to colour in this figure legend, the reader is referred to the web version of this article.)

• Among the newly detected compounds, one of the most noteworthy signals was observed in an egg sample, characterized by an *m*/*z* of 162.98256 and a RT of 1.823 min (Fig. 4a). This feature exhibited CF₂-normalized KMD values comparable to those of known PFAS belonging to the perfluoroalkyl carboxylic acids (PFCA) group (Fig. 5). Moreover, its retention time was even shorter than that of PFBA, the smallest PFCA included in our database, suggesting its structural affiliation with this compound class. An authentic reference standard enabled unambiguous identification of perfluoropropanoic acid (PFPrA, C₃HF₅O₂).

Several other egg samples exhibited a chromatographic peak at the same retention time as PFPrA (Fig. 4a), although this compound was not detected by the CD workflow, probably due to the low intensity of the peaks. PFPrA was unambiguously identified in six other egg samples from European hotspots using the SS approach, but absent from fish. Notably, this compound had already been identified in a previous egg sample during the early development of the non-targeted workflow (Jeannot et al., 2025).

To our knowledge, this is the first report documenting the occurrence of PFPrA in foodstuffs, particularly in eggs. While PFPrA is known in various environmental waters (Chow et al., 2021; Lenka et al., 2022; Liang et al., 2023), its presence in food products has not been reported so far. To our knowledge, this is the first report documenting the occurrence of PFPrA in foodstuffs, particularly in eggs, raising questions about potential contamination pathways from the degradation of other PFAS and the transfer of scPFAS into the food chain. These findings suggests that food-producing animals could be exposed to environmental PFAS contamination, either through feed or environmental vectors. Given the growing concern around scPFAS and their increasing use, these results underline the importance of including emerging contaminants in future monitoring.

• Another relevant feature was also identified in an egg as 6:2 fluorotelomer sulfonic acid (6:2 FTS) (C_8_H_5_F_13_O_3_S) based on its exact mass *m*/*z* 426.96837 and RT 5.183 min (Fig. 4b and Fig. S2). At this stage, the CF₂-normalized KMD approach (Fig. 5) was not applicable for annotation support, as no PFAS from the fluorotelomer sulfonates (FTS) family were initially included in our internal database. This compound was confirmed at the highest confidence level, with an authentic 6:2 FTS standard, and was not detected in any other samples collected from hot spot areas.

In recent years, the use of 6:2 FTS has significantly increased particularly as a replacement for long-chain legacy PFAS such as PFOS in consumer products notably as surface treatment agents for textiles or food packaging materials (Hamid et al., 2020). Its growing use has led to widespread environmental dissemination, with frequent detection in various water sources worldwide, and in various foodstuffs, such as in marine organisms (Ali et al., 2021; Lescord et al., 2015), in crustacean species such as Mysis (Ren et al., 2022), and more recently in fish and breast milk samples from Algeria (Amziane et al., 2022). These findings highlight the environmental ubiquity of this compound and the potential for bioaccumulation and human exposure through dietary intake. Known biotransformation products—specifically PFBA, PFPeA, and PFHxA (Lenka et al., 2022; LaFond et al., 2023)—were reported in environmental matrices and increasingly detected in food samples. These three compounds were among those identified in eggs via the SS workflow (as discussed in Section 3.1), underlining the need to monitor both parent and degradation products of legacy PFAS and replacement compounds.

• Another noteworthy feature was identified as Fipronil sulfone (C₁₂H₄Cl₂F₆N₄O₂S), with *m*/*z* 450.92678 and RT 7.143 min in egg samples (Fig. 4c and Fig. S3). Based on the high-quality spectral match and retention time alignment, the compound was initially assigned a confidence level of 2a according to the PFAS identification criteria proposed by to (Charbonnet et al., 2022). The CF₂-normalized KMD approach (Fig. 5) could not support the annotation, as no structurally related PFAS standards were present in the database of this study. However, this level of confidence was subsequently upgraded to level 1 following confirmation via direct injection of the analytical standard. Fipronil sulfone was detected in 85 % of eggs from private gardens, suggesting possible contamination linked to specific domestic practices These results also highlight the added value of non-targeted workflows in capturing non-conventional but highly relevant PFAS.

Fipronil sulfone is a major metabolite of fipronil, a widely used phenylpyrazole insecticide. Similar to other fipronil metabolites, it exhibits greater toxicity than its parent compound, and is known for its environmental persistance (Li et al., 2020). Its recurrent detection in various food matrices—including plant-based products, apicultural products, as well as animal-derived products—raises concerns regarding chronic dietary exposure, particularly in regions where fipronil use has been documented (Corrias et al., 2021).

• Given the detection of fipronil sulfone in several samples, a standard of the parent compound fipronil was analyzed under identical LC-HRMS conditions to determine its RT and mass-to-charge ratio (m/z) for accurate inclusion in the SS workflow. Its identification in two egg samples from European hot spots, with the highest confidence level according to (Schymanski et al., 2014), confirms its environmental persistence despite its ban in food production in 2017 (Yao et al., 2021). As with all analytes, its absence was confirmed in procedural blanks, ensuring data reliability.

Fipronil is a commonly used active substance in veterinary products for the control of fleas, lice, and ticks, which is strictly prohibited in animals intended for human consumption and can bioaccumulate in organisms (Romero et al., 2016). The illegal use of this toxic insecticide in poultry farming led to a major contamination scandal in Europe and Asia, notably involving millions of eggs (Yao et al., 2021). Moreover, fipronil residues have been reported in a wide range of food matrices, illustrating its potential for widespread contamination across the food chain (EFSA, 2018; Liang Liang et al., 2019; Yao et al., 2021; Gasparini et al., 2024).

These findings clearly illustrate the strength of the non-targeted strategy (QuEChERS + HRMS + CD) for broad-spectrum detection of fluorinated substances beyond the conventional scope. This strategy offers a more comprehensive perspective on chemical contamination applicable in future studies. This is exemplified by the detection of fipronil and its metabolite fipronil sulfone, which, although primarily used as pesticides, have recently been included in the PFAS family under the revised OECD definition (Wang et al., 2021). This evolution in classification reflects the increasing scientific and regulatory recognition of non-traditional PFAS, and highlights the importance of broad-spectrum analytical strategies capable of capturing the full diversity of fluorinated compounds potentially relevant for food safety and risk assessment.

#### 3.2.3 Application of the NTS strategy to the characterization of commercial food samples

The NTS approach presented above led to the discovery of four additional fluorinated compounds absent from our database and not routinely monitored. These compounds, detected in samples from European hot spots, raised concerns about potential consumer exposure. To further investigate this risk, the NTS strategy was extended to all food products included in the study, with a particular focus on commercially available items. Overall, PFPrA emerged as the most frequently detected PFAS, being identified in 48 % of the 40 food samples analyzed. It was detected in 3 French (100 %) and 5 Algerian (100 %) eggs, 2 French (40 %; back of cod, and alaska hake fillet) and 1 Algerian fish (10 %; bream), 4 French crustaceans (100 %), 2 French (40 %; white ham with rind, and chipolata) and 1 Algerian (20 %; beef) meat, and 1 French dairy product (33 %; goat's cheese “bûche”). This widespread occurrence across multiple food categories—regardless of their origin (Europe or North Africa) or source (commercial or hotspot)—highlights the ubiquitous presence of PFPrA in the food chain, despite it being a PFAS compound that is not routinely monitored. Given the growing concern about short-chain PFAS and their potential health implications, PFPrA should be prioritized for targeted monitoring. Strengthening surveillance efforts for this compound could provide valuable insights into emerging contamination patterns and support the refinement of risk assessment and regulatory frameworks.

Regardless of geographical origin or food category (eggs, fish, shellfish, meat, or dairy products), 6:2 FTS, Fipronil, and Fipronil sulfone were not detected in any of the commercial food samples analyzed in the present study. These findings suggest that, at this stage, widespread contamination of foodstuffs by these fluorinated compounds does not appear to be a major concern. Nevertheless, their detection in specific samples from our study highlights the importance of continued monitoring. Regular surveillance of these compounds could help improve our understanding of potential dietary exposure and provide early insights into emerging contamination trends. It is also important to communicate the potential risk of treating domestic chickens with products that may lead to residue presence in eggs, underlining the need for awareness and precaution in household poultry management.

## 4 Conclusion

This study demonstrates the added value of combining SS and NTS strategies for expanding our knowledge of fluorinated compounds. This approach was applied to a wide variety of foodstuffs from five matrix categories, two distinct geographical regions, and two sources: commercial food products and private individuals living in European contamination hotspots.These samples proved valuable for guiding prioritization efforts and played a key role in revealing PFAS beyond those currently targeted by routine monitoring. Four PFAS—confirmed with the highest level of confidence—were identified through this approach. PFPrA emerged as the most frequently detected compound, measured in nearly half of the 58 samples, regardless of food type, origin or source. This finding reinforces the global nature of PFAS contamination, underscores that short-chain compounds like PFPrA are already present across diverse regions, and emphasizes the need to integrate such emerging substances into future surveillance plans. The presence of other substances in hotspot samples underscores the broader detection capacity of the SS/NTS approach, enabling the detection of fluorinated contaminants beyond the strict regulatory definition of PFAS, which is essential for comprehensive chemical exposome characterization.

For future studies, coupling targeted sampling with advanced analytical tools like SS/NTS offers a forward-looking strategy for early detection and improved chemical risk assessment. This approach should be integrated into future food safety frameworks to help authorities anticipate emerging PFAS threats and strengthen public health protections.

Our findings underscore the importance of comprehensive contaminant monitoring not only for food safety but also within the wider framework of the United Nations Sustainable Development Goals (SDGs), especially those concerning health, water quality, and sustainable production, thereby contributing to efforts addressing climate change and environmental sustainability.

## CRediT authorship contribution statement

**Cassandre Jeannot:** Writing – review & editing, Writing – original draft, Investigation, Data curation. **Nicolas Macorps:** Investigation. **Ahmed Amziane:** Resources. **Bruno Le Bizec:** Funding acquisition, Conceptualization. **Julien Parinet:** Writing – review & editing, Validation, Supervision, Project administration, Funding acquisition, Conceptualization. **Gaud Dervilly:** Writing – review & editing, Validation, Supervision, Project administration, Funding acquisition, Conceptualization.

## Declaration of competing interest

The authors declare that they have no known competing financial interests or personal relationships that could have appeared to influence the work reported in this paper.

## Data Availability

Data will be made available on request.
