# Supplementary material for: High-resolution mass spectrometry for extended PFAS surveillance in food: combining suspect and non-targeted approaches
Source: Food Chem X. 2025 Jul 29;29:102843. doi: 10.1016/j.fochx.2025.102843 (PMC12345881; doi:10.1016/j.fochx.2025.102843)

**Supplementary data**

**Table S1.** List of PFAS reference standards available

| Family PFAS | Formula | Monoisotopic mass [M-H] | Supplier |
| --- | --- | --- | --- |
| Labelled PFAS (n = 20) | | | |
| \| PFCA \|  \| 13C4-PFBA \| \| --- \| --- \| --- \| \| PFCA \|  \| 13C2-PFHxA \| \| PFCA \|  \| 13C4-PFOA \| \| PFCA \|  \| 13C5-PFNA \| \| PFCA \|  \| 13C2-PFDA \| \| PFCA \|  \| 13C2-PFUnDA \| \| PFCA \|  \| 13C2-PFDoDA \| \| PFSA \|  \| 18O2-PFHxS \| \| PFSA \|  \| 13C4-PFOS \| \| PFSA \|  \| 13C8-PFOS \| \| PFCA \|  \| 13C3-PFPeA \| \| PFCA \|  \| 13C4-PFHpA \| \| PFSA \|  \| 13C3-PFBS \| \| FASAA \|  \| d3-N-MeFOSAA \| \| FASAA \|  \| d5-N-EtFOSAA \| \| FASA \|  \| 13C8-FOSA \| \| PFECA \|  \| 13C3-HFPO-DA \| \| FTS \|  \| 13C2-8:2 FTS \| \| FTS \|  \| 13C2-6:2 FTS \| \| diPAP \|  \| 13C4-6:2 diPAP \| | \| [13]C_4_HF_7_O_2_ \| \| --- \| \| [13]C_4_C_2_HF_11_O_2_ \| \| [13]C_4_C_4_HF_15_O_2_ \| \| [13]C_4_C_5_HF_17_O_2_ \| \| [13]C_8_C_2_HF_19_O_2_ \| \| [13]C_9_C_2_HF_21_O_2_ \| \| [13]C_10_C_2_HF_23_O_2_ \| \| C_6_HF_13_[18]OO_2_S \| \| [13]C_4_C_4_HF_17_O_3_S \| \| [13]C_8_HF_17_O_3_S \| \| [13]CC_3_HF_9_O_3_S \| \| [13]C3C_4_HF_13_O_2_ \| \| [13]CC_3_HF_9_O_3_S \| \| C_11_[2]H_3_H_3_F_17_NO_4_S \| \| C_12_[2]H_3_H_5_F_17_NO_4_S \| \| [13]C_8_H_2_F_17_NO_2_S \| \| [13]C_3_C_3_HF_11_O_3_ \| \| [13]C_8_C_2_H_5_F_17_O_3_S \| \| [13]C_6_C_2_H_5_F_13_O_3_S \| \| [13]C_12_C_4_H_9_F_26_O_4_P \| | \|  \|  \| 216.9920 \| \| --- \| --- \| --- \| \|  \|  \| 314.9780 \| \|  \|  \| 416.9790 \| \|  \|  \| 467.9790 \| \|  \|  \| 514.9670 \| \|  \|  \| 564.9640 \| \|  \|  \| 614.9590 \| \|  \|  \| 402.9450 \| \|  \|  \| 502.9430 \| \|  \|  \| 506.9560 \| \|  \|  \| 265.9860 \| \|  \|  \| 366.9820 \| \|  \|  \| 301.9520 \| \|  \|  \| 572.9850 \| \|  \|  \| 589.0140 \| \|  \|  \| 505.9720 \| \|  \|  \| 331.9778 \| \|  \|  \| 528.9682 \| \|  \|  \| 428.9746 \| \|  \|  \| 792.9885 \| | \| \| \| Wellington \| \| --- \| \| Wellington \| \| Wellington \| \| Wellington \| \| Wellington \| \| Wellington \| \| Wellington \| \| Wellington \| \| Wellington \| \| Wellington \| \| Wellington \| \| Wellington \| \| Wellington \| \| Wellington \| \| Wellington \| \| Wellington \| \| Wellington \| \| Wellington \| \| Wellington \| \| Wellington \| \|  \|  \| \| --- \| --- \| --- \| --- \| --- \| --- \| --- \| --- \| --- \| --- \| --- \| --- \| --- \| --- \| --- \| --- \| --- \| --- \| --- \| --- \| --- \| --- \| --- \| \| \| --- \| --- \| --- \| --- \| --- \| --- \| --- \| --- \| --- \| --- \| --- \| --- \| --- \| --- \| --- \| --- \| --- \| --- \| --- \| --- \| --- \| --- \| --- \| --- \| |
| PFAS (n = 45) | |  |  |
| \| PFCA \| PFBA \| \| --- \| --- \| \| PFCA \| PFPeA \| \| PFCA \| PFHxA \| \| PFCA \| PFHpA \| \| PFCA \| PFOA \| \| PFCA \| PFNA \| \| PFCA \| PFDA \| \| PFCA \| PFUnDA \| \| PFCA \| PFDoDA \| \| PFCA \| PFTrDA \| \| PFCA \| PFTeDA \| \| PFSA \| PFBS \| \| PFSA \| PFHxS \| \| PFSA \| PFHpS \| \| PFSA \| PFOS \| \| PFSA \| PFDS \| \| PFSA \| PFPeS \| \| PFSA \| PFNS \| \| PFSA \| PFUnDS \| \| PFSA \| PFDoDS \| \| PFSA \| PFTrDS \| \| FASA \| FOSA \| \| FASAA \| N-MeFOSAA \| \| FASAA \| N-EtFOSAA \| \| Cl-PFESA \| 9Cl-PF3ONS \| \| Cl-PFESA \| 11Cl-PF3UnDS \| \| PFECA \| ADONA \| \| PFECA \| HFPO-DA \| \| FTS \| 4:2 FTS \| \| FTS \| 6:2 FTS \| \| FTS \| 8:2 FTS \| \| FTS \| 10:2 FTS \| \| PFECHS \| PFECHS \| \| FTCA \| FPePA \| \| DiPAP \| 6:2 diPAP \| \| diPAP \| 8:2 diPAP \| \| FTCA \| FPrPA \| \| FTCA \| FHpPA \| \| PFESA \| PFEESA \| \| PFECA \| PFMPA \| \| PFECA \| PFMBA \| \| PFECA \| PFDHA \| \| PFCA \| PFPrA \| \| FTAB \| 6:2 FTAB \| \| FTAA \| 6:2 FTAA-Ox \| | \| C_4_HF_7_O_2_ \| \| --- \| \| C_5_HF_9_O_2_ \| \| C_6_HF_11_O_2_ \| \| C_7_HF_13_O_2_ \| \| C_8_HF_15_O_2_ \| \| C_9_HF_17_O_2_ \| \| C_10_HF_19_O_2_ \| \| C_11_HF_21_O_2_ \| \| C_12_HF_23_O_2_ \| \| C_13_HF_25_O_2_ \| \| C_14_HF_27_O_2_ \| \| C_4_HF_9_O_3_S \| \| C_6_HF_13_O_3_S \| \| C_7_HF_15_O_3_S \| \| C_8_HF_17_O_3_S \| \| C_10_HF_21_O_3_S \| \| C_5_HF_11_O_3_S \| \| C_9_HF_19_O_3_S \| \| C_11_HF_23_O_3_S \| \| C_12_HF_25_O_3_S \| \| C_13_HF_27_O_3_S \| \| C_8_H_2_F_17_NO_2_S \| \| C_11_H_6_F_17_NO_4_S \| \| C_12_H_7_F_17_NO_4_S \| \| C_8_HClF_16_O_4_S \| \| C_10_HClF_20_O_4_S \| \| C_7_H_2_F_12_O_4_ \| \| C_6_HF_11_O_3_ \| \| C_6_H_5_F_9_O_3_S \| \| C_8_H_5_F_13_O_3_S \| \| C_10_H_5_F_17_O_3_S \| \| C_12_H_5_F_21_O_3_S \| \| C_8_HF_15_O_3_S \| \| C_8_H_5_F_11_O_2_ \| \| C_16_H_9_F_26_O_4_P \| \| C_20_H_9_F_34_O_4_P \| \| C_6_H_5_F_7_O_2_ \| \| C_10_H_5_F_15_O_2_ \| \| C_4_HF_9_O_4_S \| \| C_4_HF_7_O_3_ \| \| C_5_HF_9_O_3_ \| \| C_5_HF_9_O_4_ \| \| C_3_HF_5_O_2_ \| \| C_15_H_19_F_13_N_2_O_4_S \| \| C_13_H_17_F_13_N_2_O_3_S \| | \| 213.9865 \| \| --- \| \| 263.9833 \| \| 313.9801 \| \| 363.9769 \| \| 413.9737 \| \| 463.9705 \| \| 513.9673 \| \| 563.9641 \| \| 613.9609 \| \| 663.9577 \| \| 713.9545 \| \| 299.9503 \| \| 399.9439 \| \| 449.9407 \| \| 499.9375 \| \| 599.9311 \| \| 349.9471 \| \| 549.9343 \| \| 649.9279 \| \| 699.9247 \| \| 749.9215 \| \| 498.9535 \| \| 570.9746 \| \| 583.9830 \| \| 531.9029 \| \| 631.8965 \| \| 377.9761 \| \| 329.9750 \| \| 327.9816 \| \| 427.9752 \| \| 527.9688 \| \| 627.9624 \| \| 461.9407 \| \| 342.0114 \| \| 789.9823 \| \| 989.9696 \| \| 242.0178 \| \| 442.0050 \| \| 315.9452 \| \| 229.9814 \| \| 279.9782 \| \| 295.9731 \| \| 163.9897 \| \| 570.0858 \| \| 528.0752 \| | \| Wellington \| \| --- \| \| Wellington \| \| Wellington \| \| Wellington \| \| Wellington \| \| Wellington \| \| Wellington \| \| Wellington \| \| Wellington \| \| Wellington \| \| Wellington \| \| Wellington \| \| Wellington \| \| Wellington \| \| Wellington \| \| Wellington \| \| Wellington \| \| Wellington \| \| Wellington \| \| Wellington \| \| Wellington \| \| Wellington \| \| Wellington \| \| Wellington \| \| Wellington \| \| Wellington \| \| Wellington \| \| Wellington \| \| Wellington \| \| Wellington \| \| Wellington \| \| Wellington \| \| Wellington \| \| Wellington \| \| Wellington \| \| Wellington \| \| Wellington \| \| Wellington \| \| Wellington \| \| CIL \| \| CIL \| \| CIL \| \| CIL \| \| Wellington \| \| Wellington \| |

**Table S2.** Overview of the samples analyzed by food category and geographical origin (France, Algeria, or European (EU) hot spots)

| Categories | Origin | Number | Samples |
| --- | --- | --- | --- |
| Egg (n = 21) | French | 3 |  |
|  | Algerian | 5 |  |
|  | EU hot spots | 13 |  |
| Fish (n = 20) | French | 5 | Trout fillet (n = 1), back of cod (n = 1), alaska hake fillet (n = 1), smoked salmon (n = 1) and sardine (n = 1) |
|  | Algerian | 10 | Tuna (n = 4), bream (n = 2), swordfish (n = 3) and whiting (n = 1) |
|  | EU hot spots | 5 | Eel (n = 3) and zander (n = 2) |
| Shellfish (n = 4) | French | 4 | cooked shrimps (n = 1), scallops (n = 1), raw mussels (n = 1) and cooked mussels (n = 1) |
| Meat (n = 10) | French | 5 | Butcher steak (Beef) (n = 1), cotelettes (Lamb) (n = 1), white ham with rind (Pork) (n = 1), chipolata (Pork) (n = 1) and thigh with skin (Chicken) (n = 1) |
|  | Algerian | 5 | Mutton (n = 2) and beef (n = 3) |
| Dairy products (n = 3) | French | 3 | Grated Emmental (n = 1), goat's cheese buche (n = 1) and plain cow's milk yoghurt (n = 1) |

**
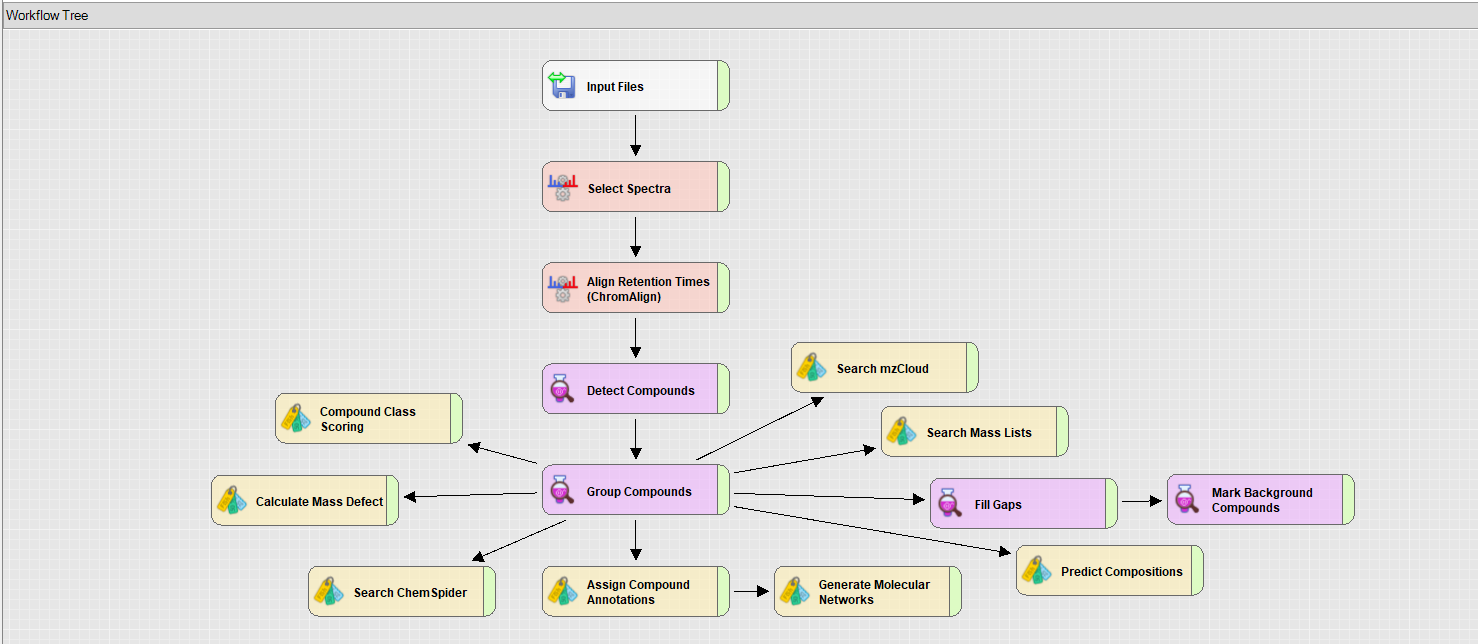
Fig. S1.** Workflow tree used in Compound Discoverer for non-targeted data analysis.

**Fig. S2.** Chromatographic profile and mass spectrum of 6:2 FTS in an egg sample from a hotspot, analyzed by LC-HRMS using the non-targeted workflow developed in this study. Signal processed with Compound Discoverer.


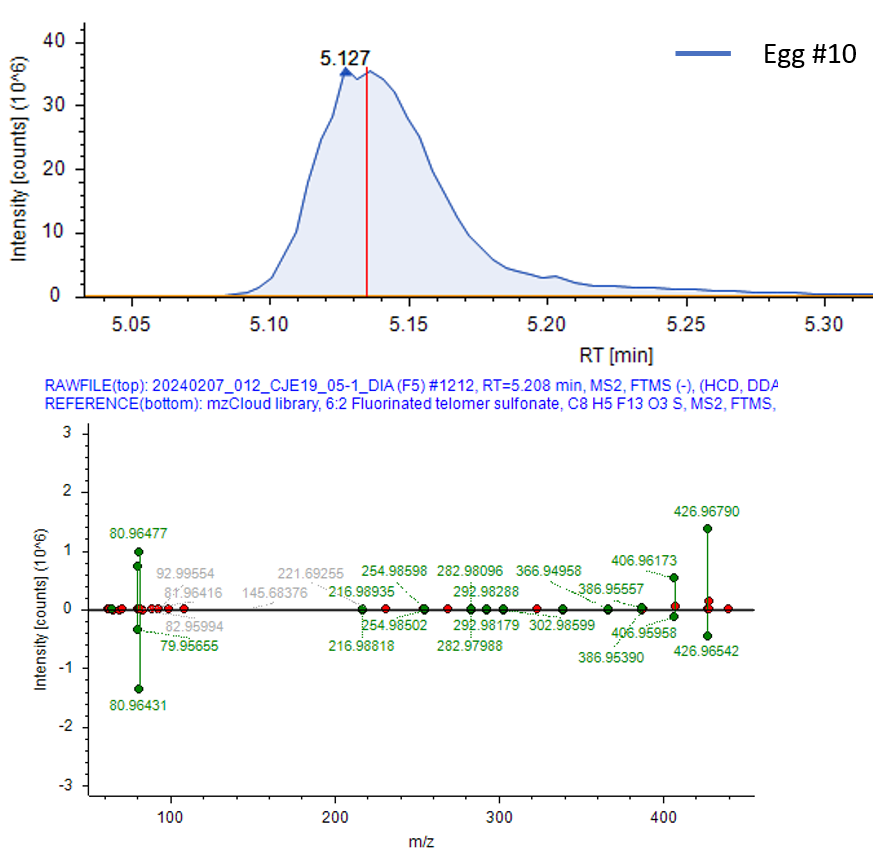


**Fig. S3.** Chromatographic profile and mass spectra of Fipronil sulfone in two egg samples from hotspot areas, analyzed by LC-HRMS using the non-targeted workflow developed in this study. Signals were processed using Compound Discoverer.


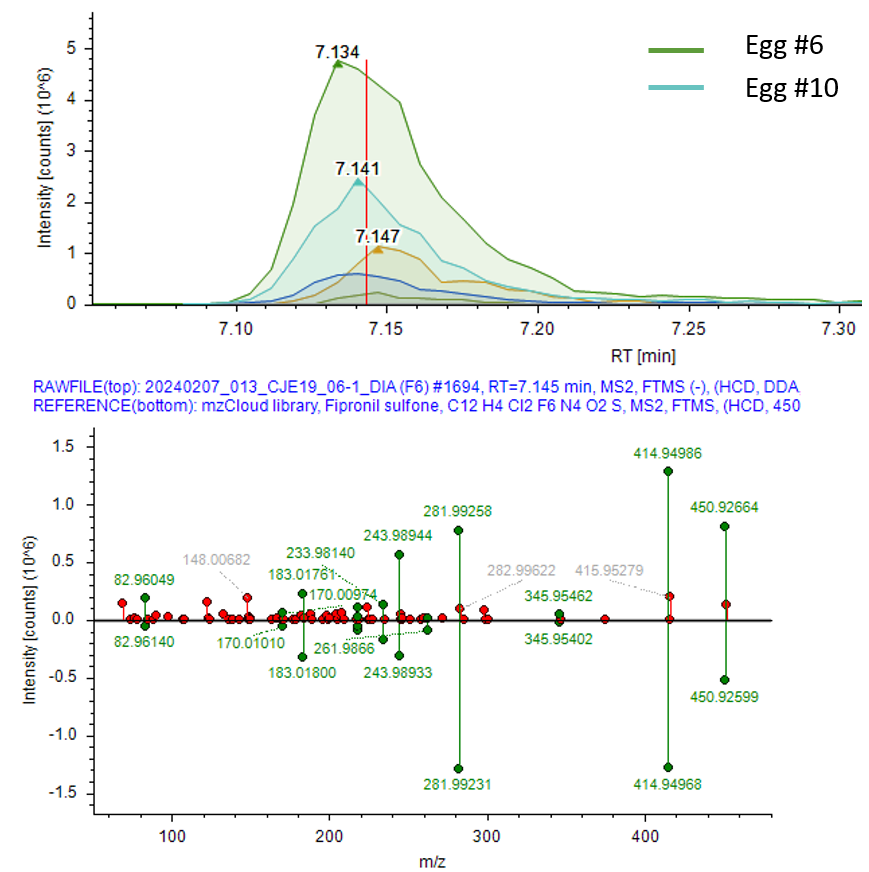

Supplement: Supplementary material [file mmc1.docx]
